# Pharmacoenvironmentology – a component of pharmacovigilance

**DOI:** 10.1186/1476-069X-6-20

**Published:** 2007-07-24

**Authors:** Syed Ziaur Rahman, Rahat Ali Khan, Varun Gupta, Misbah Uddin

**Affiliations:** 1 Department of Pharmacology, Jawaharlal Nehru Medical College, Aligarh Muslim University, Aligarh 202002, India

## Abstract

According to WHO, Pharmacovigilance activities are done to monitor detection, assessment, understanding and prevention of any obnoxious adverse reactions to drugs at therapeutic concentration on animal and human beings. However, there is also a growing focus among scientists and environmentalists about the impact of drugs on environment and surroundings. The existing term 'Ecopharmacology' is too broad and not even defined in a clear manner. The term 'Pharmacoenvironmentology' seeks to deal with the environmental impact of drugs given to humans and animals at therapeutic doses.

## Background

With growing technological advances, newer and more effective drugs are being manufactured and are used on an ever-growing scale for people with various medical conditions. Pharmacovigilance activities are done to monitor any obnoxious reactions of these drugs at therapeutic concentrations. With growing research in the field of ecology and environment, many of the adverse effects of these drugs on the environment have come to light. The first study that detected drugs in sewage took place in 1976 at the Big Blue River sewage treatment plant in Kansas City [1]. In the meantime, a number of findings related to rising levels of some drugs and their adverse effects on the flora and fauna has necessitated some action by regulatory agencies like the FDA and the European Union. Still, there lacks a substantial protocol for prospective monitoring of drug concentrations in the environment and the evident adverse effects.

## Discussion

We are living in an environment that is polluted not only by heavy metals, pesticides, and emissions from gasoline engines, but also with pharmaceutical chemicals. These pharmaceuticals enter into the environment through various routes causing harmful effects.

Literature on the above subject was searched in almost all major search engines including PubMed with the keywords – Ecopharmacology, Pharmaceuticals and Personal Care Products (PPCPs), Clinical trials and Environment. A number of studies measuring the levels in surface water, groundwater and drinking water of some drugs given therapeutically to humans and animals including antibiotics, hormones, pain killers, tranquilizers, beta blockers and anticancers were found [2-8]. Development of antibiotic resistance in pathogens in the environment owing to their exposure was the major concern. Richard and Cook even proposed a mechanistic approach to the exposure analysis for environmental risk assessment [9]. Some prominent examples of drugs causing harmful effects on environment are that of vultures' death after consuming carcasses of animals treated with Diclofenac sodium [10-12], Ethinyl estradiol adversely affecting fish through its "feminization" of males [13], antidepressant drugs like Fluoxetine (Prozac) triggering spawning in shellfish and traces of Cocaine detected in River Thames [14]. A few drugs are so synthesized that they tend to persist in the environment even after their excretion. Clofibric acid in the aquatic environment disturbing the local fauna is an example.

When a human or animal is given a drug orally, it may either be fully or poorly absorbed from the gastrointestinal tract. Clearly, unabsorbed drug will pass into the environment along with faeces. When humans or animals are given drugs parenterally or orally, the drug may be metabolized to a greater or lesser extent and excreted into the environment (including in exhaled air) as parent drug or metabolites, or as a mixture of both. It means that once they are excreted into the environment, they enter food chains and concentrate as they move upward into larger predators [15]. Ecopharmacology (Ecosystem + pharmacology) describes entry of chemicals or drugs into the environment through any route and at any concentration disturbing the balance of ecology (ecosystem), as a consequence. If these drugs enter through living organisms via elimination subsequent to pharmacotherapy, it should be a specific domain of pharmacology and not of environmental studies. This domain may be referred as Pharmacoenvironmentology. Apart from that, Ecopharmacology as a major term should be restricted to studies of "PPCPs" irrespective of doses and route of entry into environment. PPCPs comprise a very broad and diverse collection of groups of chemicals substances comprising all human and veterinary drugs (available by prescription or over-the-counter; including the new genre of "biologics"), diagnostic agents, "nutraceuticals" (bioactive food supplements), and other consumer chemicals, such as fragrances, cosmetics and sun-screen agents, "excipients" (so-called "inert" ingredients), biopharmaceuticals, dyes, pesticides, and many others [16]. This broad collection of substances refers, in general, to any product consumed by individuals for personal health or cosmetic reasons. The term Pharmacoenvironmentology can be used for this specialty dealing specifically with pharmacological agents and their impact on the environment, after elimination from humans and animals as post-therapy.

Though a number of regulatory bodies like the FDA and the European Union have set some cut-off limit for environmental concentration of drugs, no actual testing is conducted after a drug is marketed to see if the environmental concentration was estimated correctly.

When a new drug is proposed for market, FDA requires the manufacturer to conduct a risk assessment that estimates the concentrations that will be found in the environment. If the risk assessment concludes that the concentration will be less than one part per billion, the drug is assumed to pose acceptable risks. FDA has never turned down a proposed new drug based on estimated environmental concentrations, and no actual testing is conducted after a drug is marketed to see if the environmental concentration was estimated correctly [17]. Apart from that there is little concern and research to find the adverse effects on environment, of particular drugs given at therapeutic doses. Even in clinical trials, where many limitations like that of limited size, narrow population, narrow indications and short duration are observed, we also found that evaluation of drugs on environment is practiced very minimally.

The European Union has described a two-phased approach to evaluate Medicinal Products in environment. The environmental concentration of the medicinal product is calculated in Phase I. Substances with a very high specific mode of action like hormones are directed to Phase II irrespectively of the result of the exposure calculation. In the second phase, information on the physical, chemical and toxicological properties are obtained and assessed in relation to the environmental exposure of the medicinal product [18]. Similarly, environmental risk assessments are also an integral part of the assessment process in the granting of marketing authorizations for veterinary medicinal products [19]. According to John P. Sumpter (2007), these recent European Medicines Agency guidelines covering the environmental risk assessment of human pharmaceuticals are a step in the right direction, but a more sophisticated approach, rather than a "one-fits-all" solution, is probably needed [20]. As a part of a Good Clinical Trial, studies on impact of particular drugs on the environment should too be incorporated. Some concerns that need to be taken up under Pharmacoenvironmentology are that of drugs and their exact concentration in different components of the environment.

## Conclusion

Pharmacovigilance pertains to the activities of adverse effects of drugs at therapeutic doses on animal and human beings. In this context, Pharmacoenvironmentology may be an extension of Pharmacovigilance dealing specifically with the effects pertaining to the environment and ecology of drugs given in therapeutic concentrations. Pharmacologists having this particular expertise (pharmacoenvironmentologist) may be made a compulsory component of the team assessing different aspects of drug safety. We need to monitor the effects of drugs not only as a good medical practice, but also to safeguard our environment.

## Competing interests

Authors do not have any conflict of interest with any organization both personally and financially. Thus, the authors declare that they have no competing interests.

## Authors' contributions

All authors have made substantial contributions while writing this paper right from conception and design, acquisition of data and finally in the analysis and interpretation of the data. All authors have also been involved in drafting the manuscript and revising it critically for important intellectual content. We give final approval of the version to be published. Each author has participated sufficiently in the work to take public responsibility for appropriate portions of the content. All authors have read and approved the final manuscript.
